# A novel approach to determining augmented bone volume in intraoral bone block augmentation using an intraoral scanner: an in vitro study

**DOI:** 10.1186/s40729-023-00492-0

**Published:** 2023-09-05

**Authors:** Weichel Frederic, Kalchthaler Lukas, Kühle Reinald, Büsch Christopher, Ristow Oliver, Engel Michael, Freudlsperger Christian, Hoffmann Jürgen, Mertens Christian

**Affiliations:** 1 Department of Oral‐ and Cranio‐Maxillofacial Surgery, Heidelberg University Hospital, Im Neuenheimer Feld 400, 69120 Heidelberg, Germany; 2 Institute of Medical Biometry, University Heidelberg, Im Neuenheimer Feld 130.3, 69120 Heidelberg, Germany

**Keywords:** Bone augmentation, Bone regeneration, Autogenous bone, Bone blocks, intraoral bone graft, Automated volume measurement

## Abstract

**Introduction:**

Bone augmentation procedures are established tools for reshaping the alveolar ridge and increasing bone volume. Different approaches are being used to measure postoperative bone volume gain. This study aimed to develop an objective and automated volume measurement tool equally as precise as manual slice-by-slice annotation.

**Materials and methods:**

To evaluate the proposed workflow, we performed an in vitro study with 20 pig mandibles that were grafted using three different grafting techniques—autogenous full block, split block bone and shell augmentation. The pig jaws were scanned pre- and postoperatively using an intraoral scanner. The resulting surface files (baseline, full block, split block, shell) were processed using the new volume-measuring workflow as well as using manual slice-by-slice annotation at baseline (*t*0) and at 6 months (*t*1) using the same population. Two TOSTs (Test of One-Sided Significance) and NHSTs (Null Hypothesis Significance Test) were used to compare the two workflows. The intra-rater reliability between *t*0 and *t*1 was determined using intraclass correlation coefficients.

**Results:**

The mean difference for the full block augmentation technique was − 0.015 cm^3^ (*p* < 0.001); for the split block technique, it was − 0.034 cm^3^ *p* = 0.01, and for the shell technique, it was − 0.042 cm^3^. All results were statistically not different from zero and statistically equivalent to zero. The results also showed an excellent absolute intra-rater agreement.

**Conclusions:**

The semiautomatic volume measurement established in this article achieves comparable results to manual slice-by-slice measuring in determining volumes on STL files generated by intraoral scanners and shows an excellent intra-rater reliability.

**Graphical Abstract:**

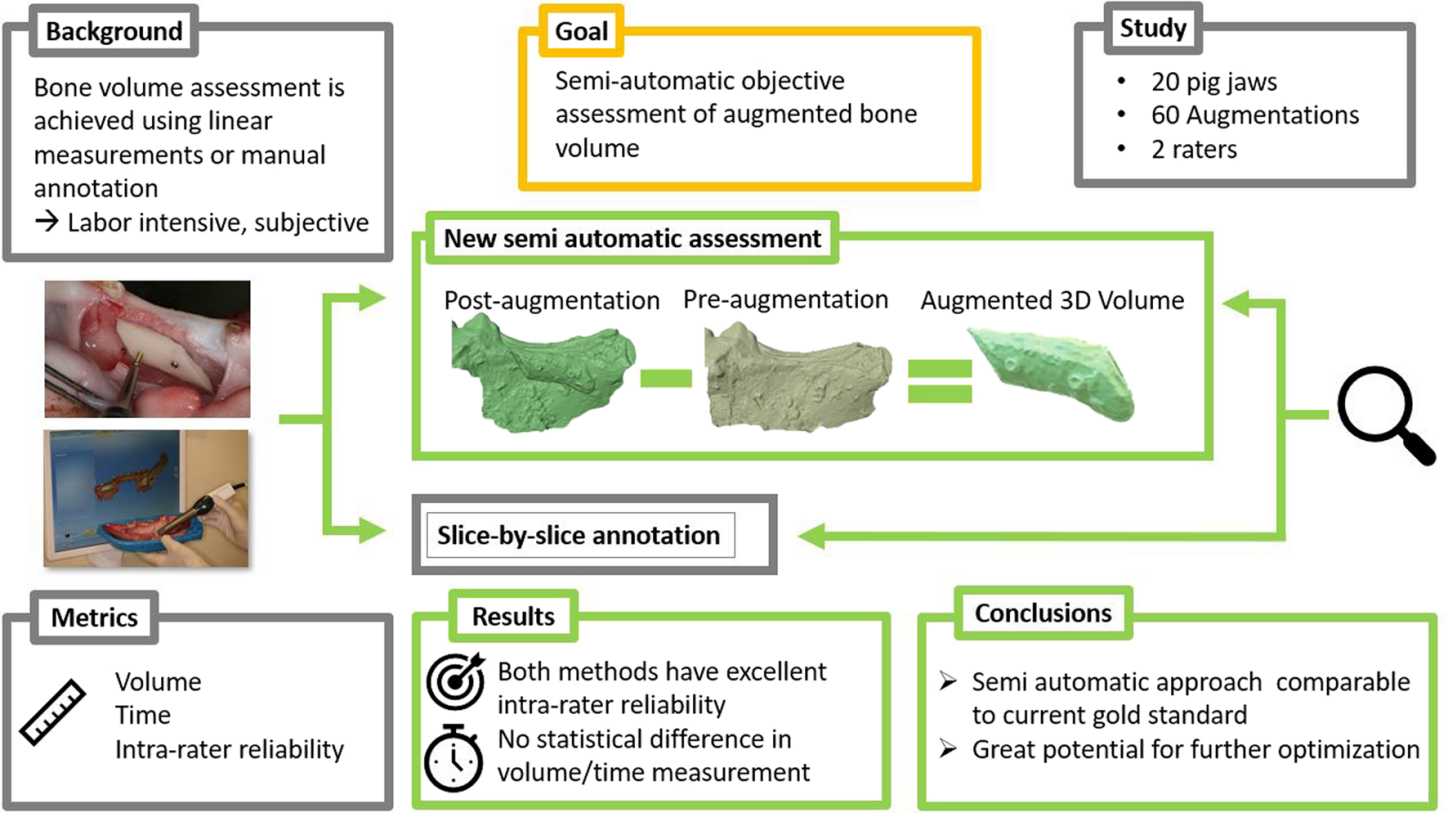

## Introduction

Bony defects may prevent the placement of dental implants. Different bone augmentation techniques can be employed to alleviate this problem by increasing the bone availability at the implantation site [1, 2].

The success of such procedures is generally quantified radiologically, based on either a two-dimensional or a three-dimensional radiograph. However, the three-dimensional procedures often involve only a single measurement at specific points along the alveolar ridge [3]. These do not adequately represent the actual volume gain. Precise three-dimensional volumetric measurements, however, require labour-intensive image annotation [4–6]. In recent years, augmentation analysis using a threshold-based segmentation approach was established for maxillary sinus augmentation [7]. Hence, we wanted to develop an algorithm to perform three-dimensional analysis along the alveolar ridge which is less labour intensive and more precise than the current standard.

Furthermore, we wanted to establish an algorithm that would not only allow the use of radiographic data for analysis, e.g., CBCT or CT scans, but also allow the use of three-dimensional data acquired by intraoral scanners. This flexibility would enable the algorithm to be used in vitro as well as in vivo to study bone augmentation volume. Furthermore, this algorithm could also be used to evaluate the outcome of soft-tissue grafting procedures or alveolar ridge remodelling after tooth extraction.

Therefore, we aimed to develop an automated and reliable three-dimensional measurement technique to quantify the volume gain after bone augmentation procedures. We show the in vitro application of this new semi-automatic algorithm in evaluating three different bone grafting procedures and compare it to the established manual segmentation workflow.

The primary outcome of this study was the difference in the measured bone volume, with secondary outcomes being the time used for the measurement process as well as the intra-rater reliability. In our statistical equivalence assessment, the null hypothesis is the presence of a true effect or of an effect that is worth examining.

## Materials and methods

In this in vitro study, the volumetric analysis was performed on 20 pig mandibles. Each site was scanned prior to bone grafting to obtain a baseline intraoral scan, and was then used for three different consecutive autologous bone augmentation procedures in the premolar region. After each grafting procedure intraoral scans (Omnicam, Dentsply Sirona, Bensheim, Germany) were performed.

### Surgical procedure

Bone blocks were harvested from the retromolar region using the microsaw protocol (Dentsply Sirona) or a trephine drill.

The recipient site was prepared by a crestal incision and vertical releasing incisions. A mucoperiosteal flap was elevated.

**Fig. 1:**
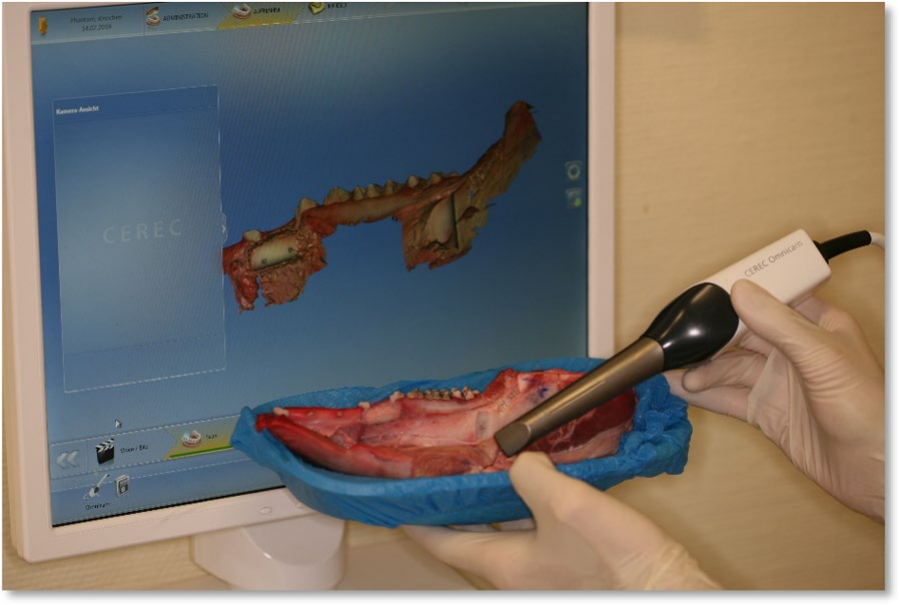
Intraoral scan. The process of scanning the pig mandible is shown. On the screen, the already scanned 3D object is visualized with colour information. In the foreground, the surgeon moves the scan head across the pig mandible to capture all necessary aspects

For the first group, the elevated full block (B) was placed in such a manner that bony contact was maximized. The block was fixed with two titanium screws. An intraoral scan was performed to document the augmentation result (Fig. 1).

For the second group, a split block technique (S1) was used, meaning that the titanium screws were removed, and the bone block was thinned out to ~ 1 mm thickness using a bone scraper (Safescraper Twist, META, Reggio Emilia, Italy). At the defect site, the thinned-out cortical plate was secured at ~ 5 mm distance from the alveolar crest with two osteosynthesis screws. The particulate bone material was then packed into the gap between cortical plate and the defect site. A third intraoral scan was performed.

For the third group, the titanium screws, bone block S1 and particulate bone material were removed. A bone block was elevated using a large diameter trephine drill (S2). The resulting curved bone shell was fixed with two titanium screws at ~ 5 mm distance from the alveolar crest. The resulting gap between the bony shell and alveolar bone was then filled with particulate bone material, which was packed into position. A final intraoral scan was performed.

### STL format and object registration

Commercially available intraoral scanners generate STL 3D objects in the STL (surface triangle language) file format. This format consists of many vertices that are connected to triangles which are combined with a normal vector to form the surface model. Each scan exists in its own reference frame and is not aligned to other scans in a meaningful way. In both methods, the first step is the alignment of the scans after augmentation (B, S1 and S2) onto the preoperative intraoral scan. This process is called registration and is performed using a three-point alignment.

### Manual measurement: the current gold standard

After alignment of the four scans, it was possible to manually trace the difference between the preoperative B and the S1 or S2 scan in a slice view. A slice thickness of 0.25 mm was used. These annotated slices were combined to create the 3D volume which represented the augmented bone volume. The complete volume could then be calculated in cm^3^. This analysis was performed using implant-planning software (Simplant Pro 18.0, Dentsply Sirona Implants, Hasselt, Belgium).

### Semiautomatic measurement: proposed algorithm

The necessary steps to determine the model volume using our proposed measuring method are as follows:

1. Refined registration.
2. Deletion of superfluous vertices.
3. Closing of surface mesh to a volume mesh.
4. Definition of the region of interest (ROI) as a volume mesh.
5. Creation of new volume meshes out of the overlapping volume of the ROI mesh and volume meshes of the augmented jaws (one new volume mesh each for augmentation B, S1 and S2).
6. Subtraction of the initial, not augmented, jaw segment from the augmented jaw segments.
7. Deletion of any unconnected volumes.
8. Calculation of the remaining volume in cm^3^.

The open-source software “Blender v2.38” (Community, B.O., 2018. *Blender—a 3D modelling and rendering package*, Stichting Blender Foundation, Amsterdam) was used.

### Refined registration

**Fig. 2:**
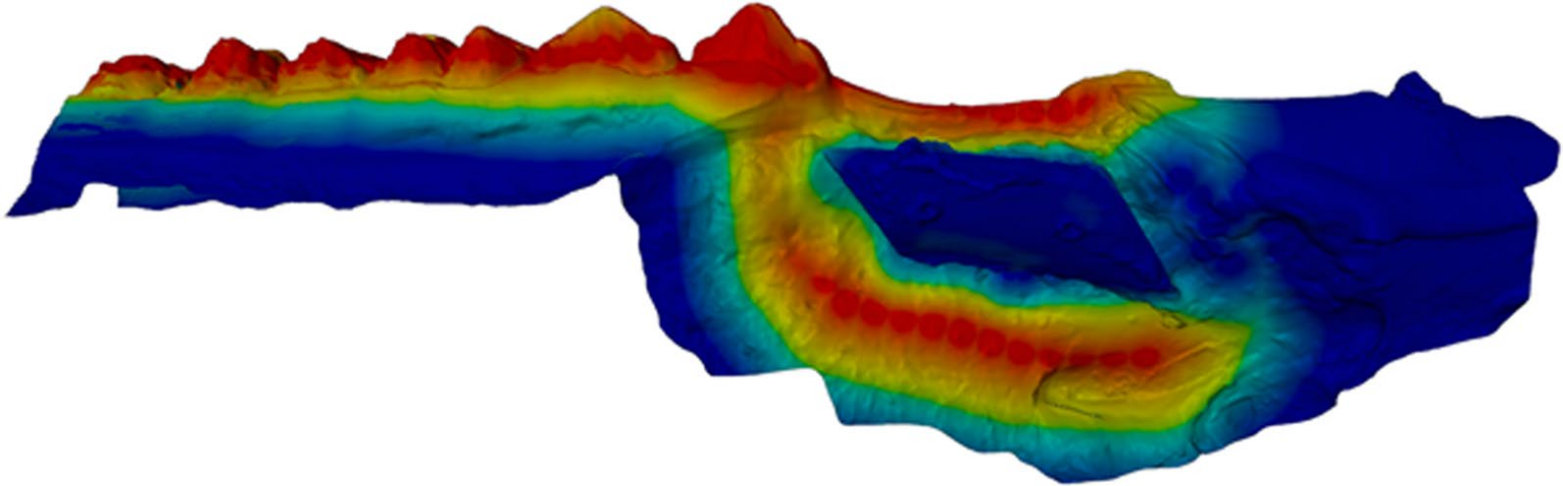
Weight map of the ICP-algorithm. To achieve the best alignment between the different surface models (one for each augmentation technique), stable region surface models were highlighted. These highlighted areas were then used for the ICP-algorithm. The colours correspond to the relative weight in the ICP-calculation from red (most important) to blue (not used for alignment)

**Fig. 3:**
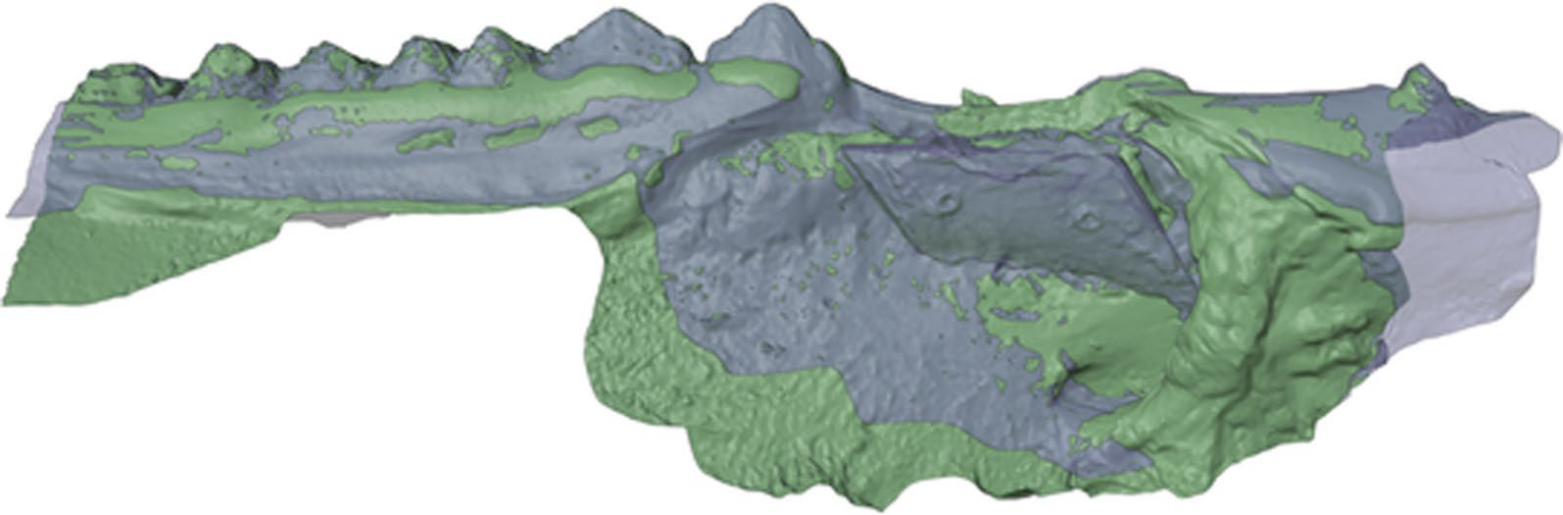
Aligned scan of full block augmentation. After the ICP alignment, the surface models were overlapping each other. The augmented volume of the full block augmentation (blue transparent) protruded beyond the base scan (green)

All intraoral scans of one pig mandible were aligned using a free and open-source software plugin for blender [8]. After the three-point registration, a weighted iterative closest point (ICP) matching using the Kabsch algorithm was applied [9] to improve the alignment. The weighted ICP algorithm allows selection of a specific area of the surfaces to perform the matching (Fig. 2). Thus, the augmented bone volume could be excluded in the registration. Furthermore, only stable, bony parts of the surface scan were selected for the alignment. After the ICP registration, the quality of the matching was visually confirmed (Fig. 3).

### Deletion of superfluous vertices

**Fig. 4:**
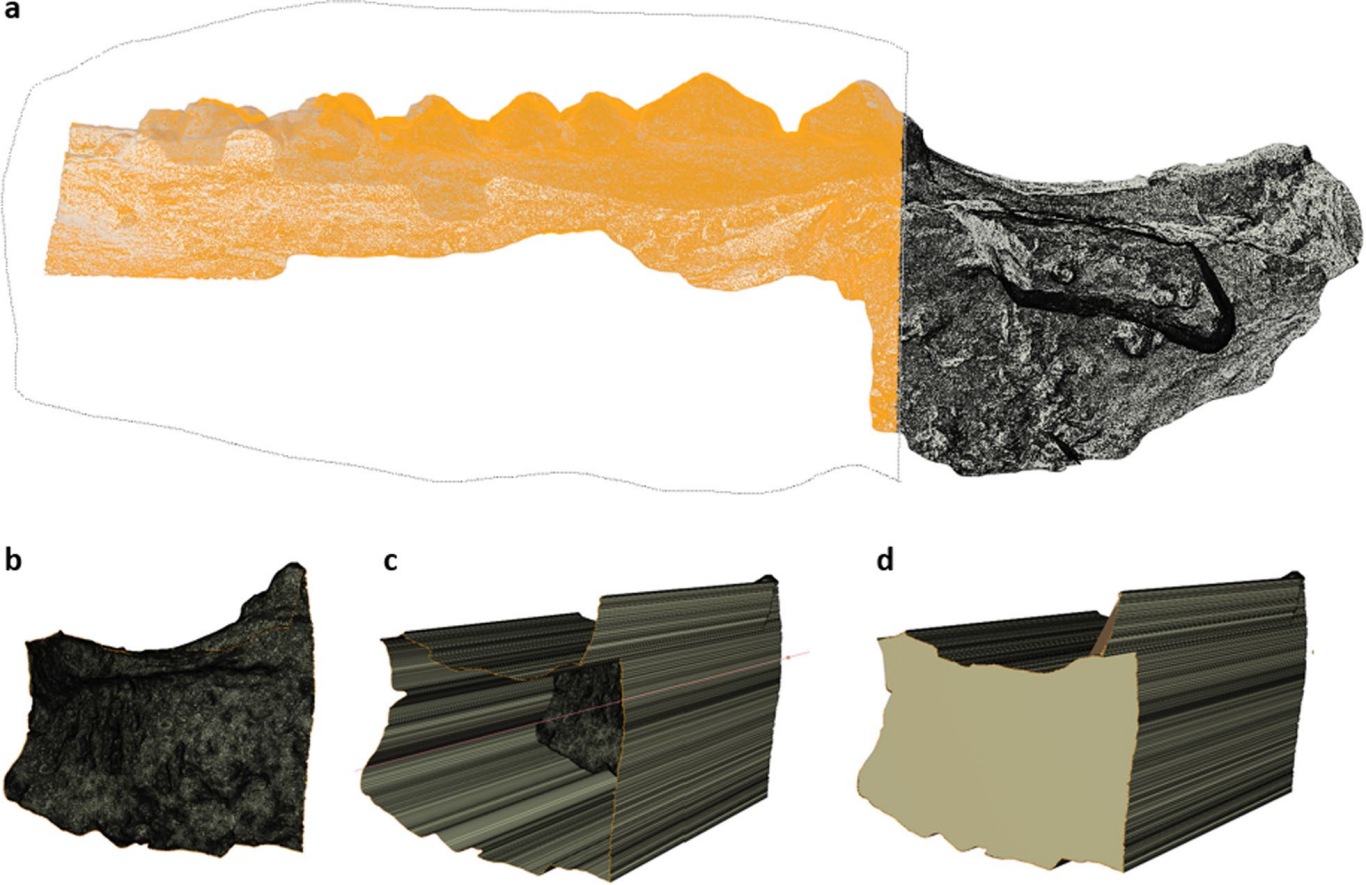
Postprocessing of the intraoral scan. Important steps in postprocessing of the intraoral scan are shown. **a** On the right side of the object, the full block augmented area can be seen (black). On the left, a loop-select tool (dotted line) is used to select the area of the scan which is not necessary for volume measurement (orange) so it can be deleted. **b**–**d** Process of giving the surface model a volume by extruding the border of the 3D model and finally closing the open face is shown

To select the ROI and measure the volume difference, only a subsection of the complete intraoral scan is necessary. In this step, unnecessary vertices were removed to facilitate further file processing (Fig. 4a).

### Closing of surface mesh to a volume mesh

After the previous step, the intraoral scan should only consist of a mesh which shows one general face on which the augmented region is situated. Then, the “loop select tool” is used to select the border of the mesh and extrude it perpendicularly to the mesh face, giving it depth. Using the “Fill” tool created a triangulated new face closing the mesh (Fig. 4b–d).

### Definition of the region of interest (ROI) as a volume mesh

**Fig. 5:**
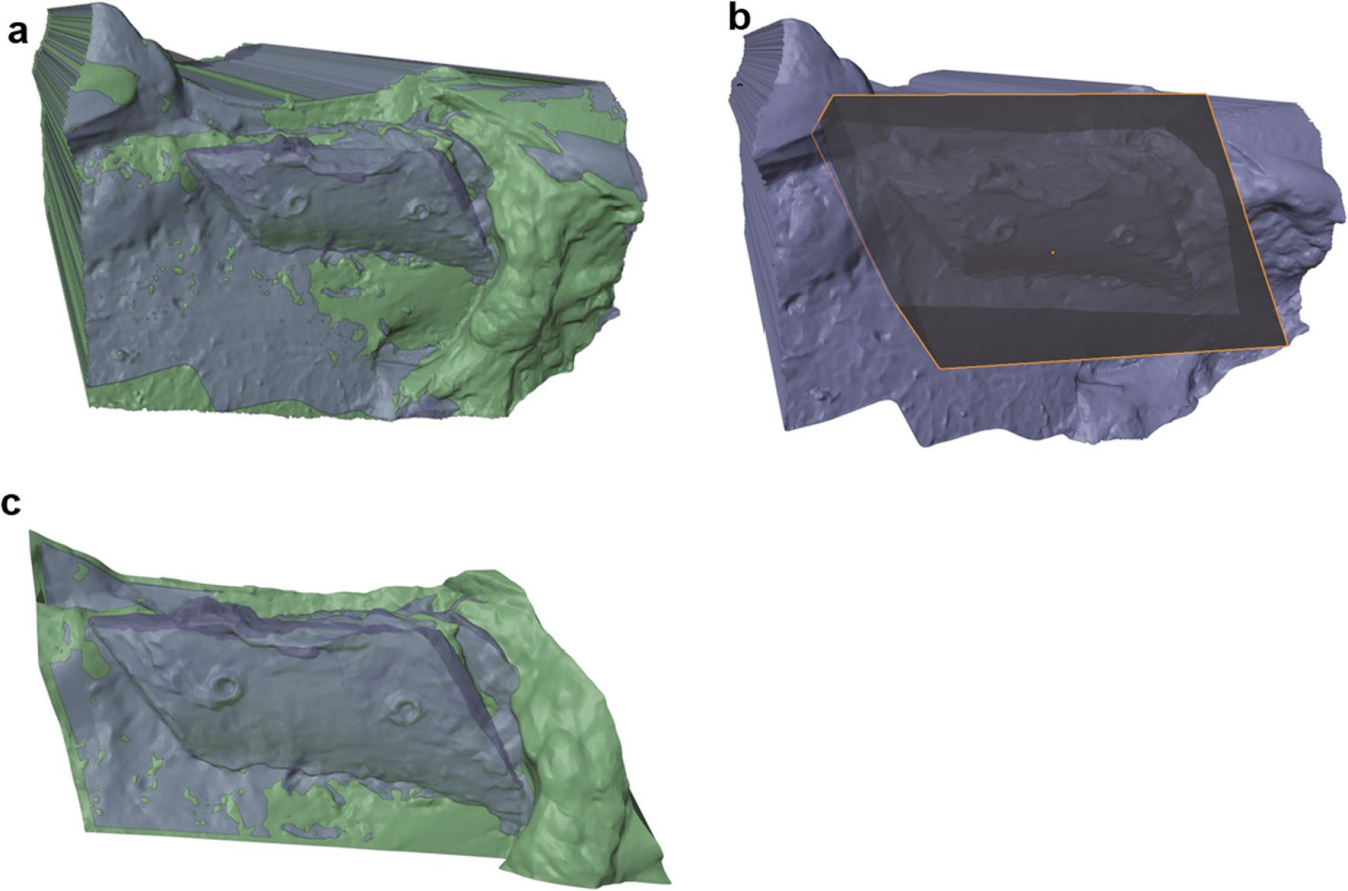
Selection of the region of interest. **a** Aligned scan of full-block augmentation as a closed object (blue transparent) and ground truth (green). **b** Region of interest is defined using a cube object (grey transparent) which includes the augmentation with a safety margin. **c** Intersection of the region of interest cube from that shown in **b** and the augmentation surface scan is calculated

The ROI can be defined using a cube or any other 3D object, which should be manipulated in such a way that it includes the whole augmented region. The final ROI Object (ROI 1) needs to be duplicated and slightly scaled down resulting in a second ROI Object (ROI 2). Now, an intersection mesh between ROI 1 and the baseline model, and between ROI 2 and the augmented models can be calculated. The usage of two ROI objects of different sizes is necessary to enable subtraction of the baseline object from the augmented objects (Fig. 5).

### Calculation of the augmented meshes

**Fig. 6:**
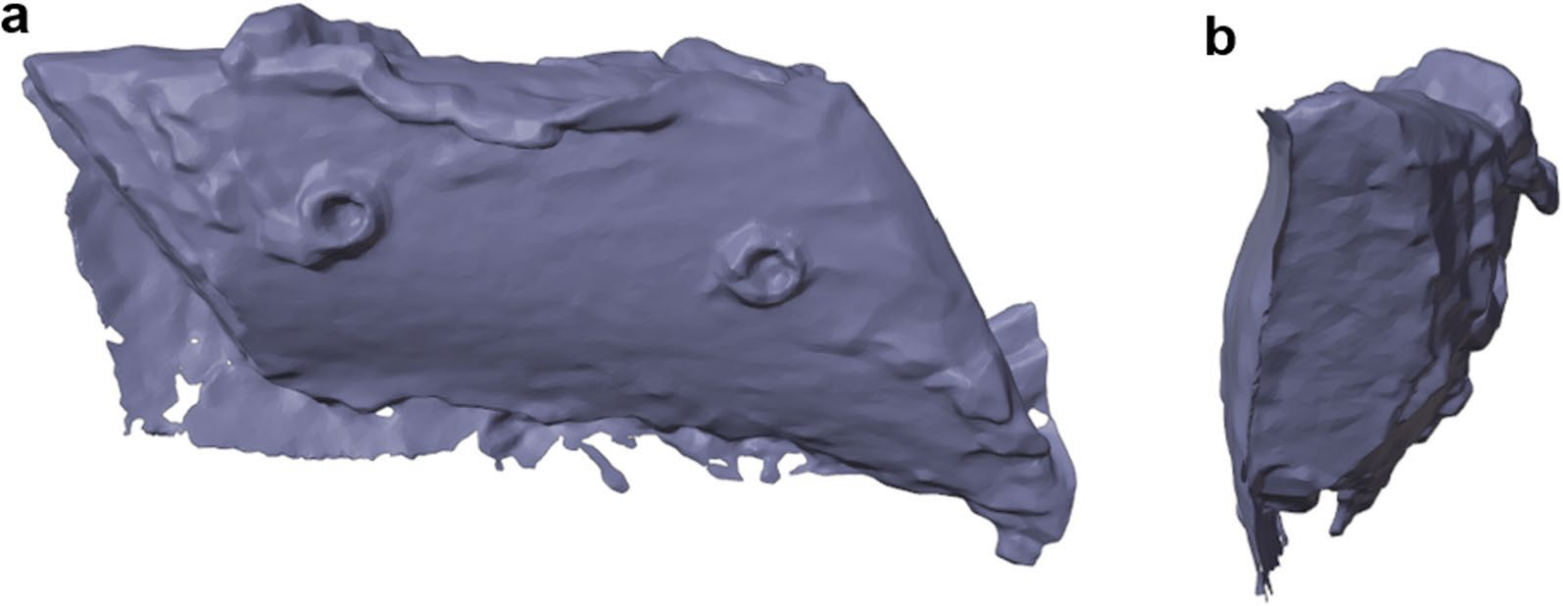
3D model of the augmented bone volume. After subtraction of the base scan the resulting 3D model comprised the augmented bone volume shown in frontal (**a**) and lateral (**b**) view

Finally, the models which only contain the ROI from the previous step were used to calculate the 3D object of the augmented region. This was achieved by subtracting the mesh of the baseline scan from the augmented meshes (Fig. 6). This calculates augmented bone volume (Boolean subtraction). Especially, when working with particulate bone grafting material, e.g., in techniques S1 and S2, small particles which do not belong to the main augmented region can still remain after this step. Because they are generally not attached to the main volume and are free floating, any such disconnected parts of the main augmented region can be deleted, and the volume of the remaining 3D object can be calculated.

### Data acquisition

Measurements were performed by one dental surgeon using the manual segmentation approach and one using the semiautomatic approach. An intraoral scan of the pig mandible was performed once immediately after each augmentation procedure. The resulting STL files were analysed twice, once at t0 and then again at *t*1, 6 months later, using the algorithms described above. The assessors were not involved in the surgical treatment.

### Time measurement

To compare both algorithms in terms of their labour intensity, we measured the time beginning after loading the STL files in the respective programs and measuring until the volume measurement was completed.

### Statistical methods

Continuous variables are expressed as mean ± SD. In addition, the number of non-missing values was added.

Equivalence tests were performed to examine whether the automated bone volume determination procedure was equivalent to the manual bone volume determination procedure (objective of the trial). To analyse the difference between the two bone volume determination procedures the (automated blender–manual-orthogon) calculation was used. Hence, a value of zero indicated that there was no difference between the two bone volume determination procedures. The equivalence test was performed with TOST (Test of One-Sided Significance), meaning that the null hypothesis is the presence of a true effect and the alternative hypothesis is an effect, for which the 90% confidence interval falls within the equivalence bounds. The margin of a meaningful effect for the upper and lower equivalence bounds for the two one-sided tests was set to 0.15 cm^3^, which Arasawa et al. already stated should be considered as satisfactorily accurate [7]. In addition, NHST (Null Hypothesis Significance Testing) was performed (*t* tests).

Moreover, intraclass correlation coefficients (ICC) with corresponding 95% confidence intervals were computed to assess the test–retest/intra-rater reliability of the volume calculations at two different timepoints (*t*0 and *t*1) using a two-way mixed effect model with single measurements. Interpretation provided by Koo and Li was used for ICC value interpretation: < 0.5 = poor, 0.5–0.75 = moderate, 0.75–0.9 = good and < 0.9 = excellent reliability [10].

To investigate whether the total time needed for measurement following each augmentation technique (block, split block and shell) between the automated blender and manual-orthogon methods was different, Wilcoxon two-sample signed-rank tests were performed, because the data were paired and not normally distributed. Furthermore, boxplots were generated for illustration purposes.

Due to the design and small sample size of the study, the analyses are purely of a descriptive nature; all *p* values need to be interpreted in a descriptive sense and have no confirmatory value. A *p* value smaller than 0.05 was considered statistically significant. Statistical analyses were conducted in R (version 4.1.2, R Core Team, Auckland, New Zealand) using the packages “TOSTER” for equivalence tests and “irr” for ICC calculations.

## Results

Block augmentation measured at t1 with the manual method resulted in an augmented volume of 0.35 cm^3^ ± 0.085. The automated measuring method determined the volume to be slightly lower at 0.34 cm^3^ ± 0.091. This resulted in a mean difference of − 0.015 cm^3^ (TOST: 90% CI [− 0.062;0.033], *p* < 0.001) compared with the manual method.

For the split block augmentation (S1) at t1, the mean volume was determined to be 0.78 cm^3^ ± 0.15 using the manual method and 0.75 cm^3^ ± 0.15 with the semiautomatic method. The mean difference for the split block technique was, therefore, found to be − 0.034 cm^3^ (TOST: 90% CI [− 0.115; 0.046], *p* = 0.010).

**Fig. 7:**
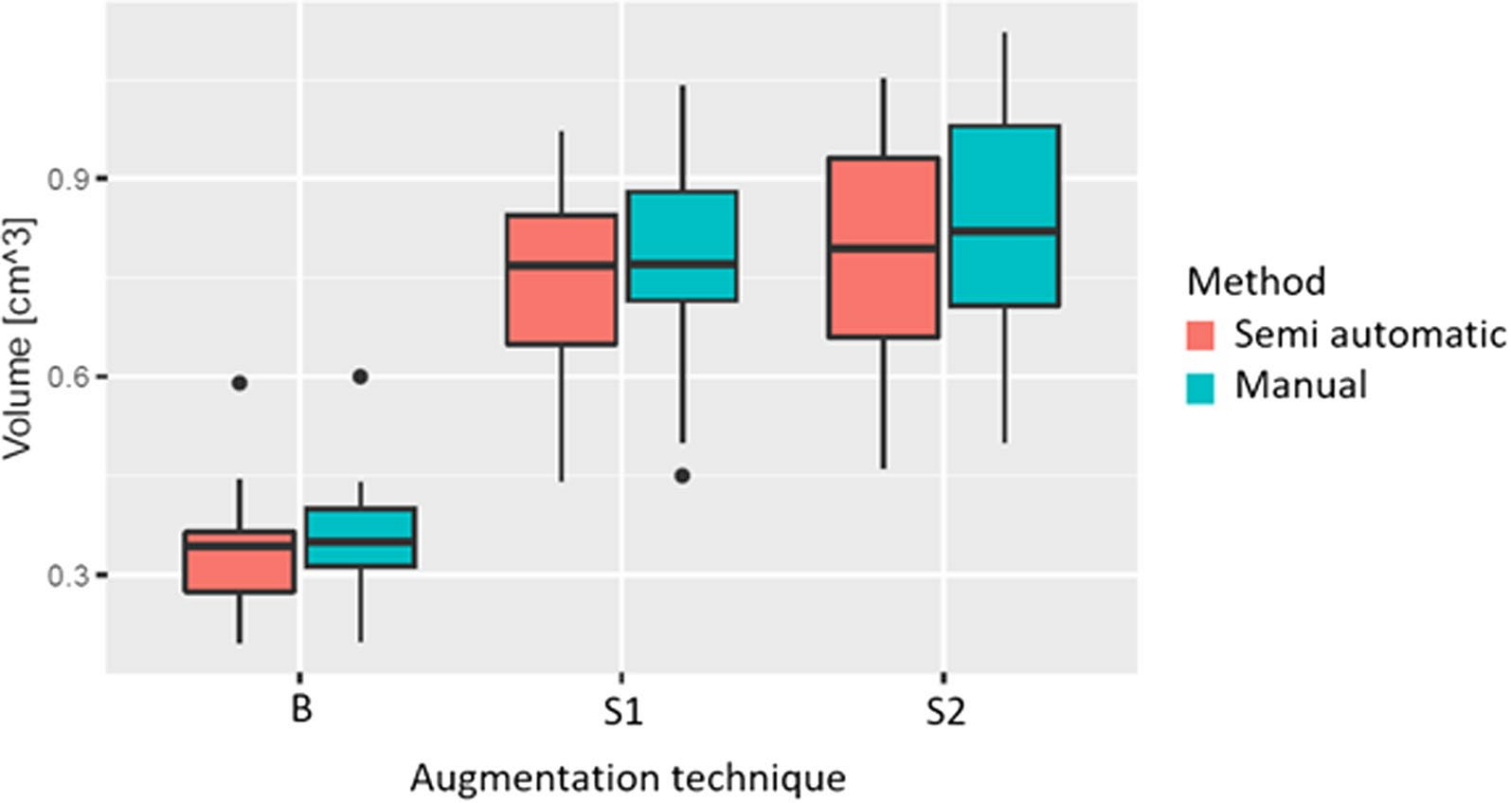
Absolute values of augmented bone volume measured using each technique. Comparison of the measured volumes of the proposed semi-automatic approach with the manual slice-by-slice technique. Shown are boxplots of the values of the augmented bone volume for the different augmentation techniques (B: block augmentation, S1: split-block augmentation, S2: shell)

For the shell augmentation (S2) at *t*1, the mean volume was 0.84 cm^3^ ± 0.16 using the manual approach and 0.80 cm^3^ ± 0.18 using the semiautomatic approach. This resulted in a mean difference of − 0.042 cm^3^ (TOST: 90% CI [− 0.134; 0.051], *p* = 0.028). All results were statistically not different from zero and statistically equivalent to zero (Table 1; Fig. 7).

**Table 1:** Volume measurements

	Time-point	Semi-automatic (*N* = 19) [cm^3^]	Manual (*N* = 20) [cm^3^]	Total (*N* = 39) [cm^3^]	Mean difference [90% Confidence Interval]	*p* values (TOST, NHST)
Block augmentation	T0	0.35 ± 0.081	0.36 ± 0.091	0.35 ± 0.085	− 0.008 [− 0.054, 0.038]	< 0.001*, 0.773
	T1	0.34 ± 0.091	0.35 ± 0.085	0.35 ± 0.087	− 0.015 [− 0.062, 0.033]	< 0.001*, 0.609
Split block augmentation	T0	0.73 ± 0.15	0.78 ± 0.14	0.75 ± 0.14	− 0.05 [− 0.127, 0.027]	0.017*, 0.280
	T1	0.75 ± 0.15	0.78 ± 0.15	0.76 ± 0.15	− 0.034 [− 0.115, 0.046]	0.010*, 0.475
Shell augmentation	T0	0.80 ± 0.19	0.86 ± 0.17	0.83 ± 0.18	− 0.066 [− 0.161, 0.029]	0.073, 0.248
	T1	0.80 ± 0.18	0.84 ± 016	0.82 ± 0.17	− 0.042 [− 0.134, 0.051]	0.028*, 0.452

The measured values of augmented volume for the different augmentation techniques, time points and measurement techniques. TOST: Test of One-Sided Significance, NHST: Null Hypothesis Significance Testing

*Statistically significant with significance level alpha < 0.05

One pig jaw could not be analysed using the semiautomatic approach resulting in a sample size of 19 for the semiautomatic and 20 for the conventional approach. This was due to the margin of the intraoral scan being too close to accurately calculate the volume difference.

The intra-rater reliability between *t*0 and *t*1 was determined to show excellent reliability for the manual as well as the semiautomatic method. For the block augmentation, an ICC of 0.9559 [0.894, 0.982] for the manual method and 0.9598 [0.882, 0.985] for the semiautomatic approach was observed. The ICC for the split block augmentation was 0.986 [0.965, 0.994] for the manual method and 0.9507 [0.874, 0.981] for the semiautomatic method. Finally, the measurements for the block augmentation S2 using the manual method showed an ICC of 0.9594 [0.858, 0.986] and 0.9664 [0.915, 0.987] for the semiautomatic method.

**Fig. 8:**
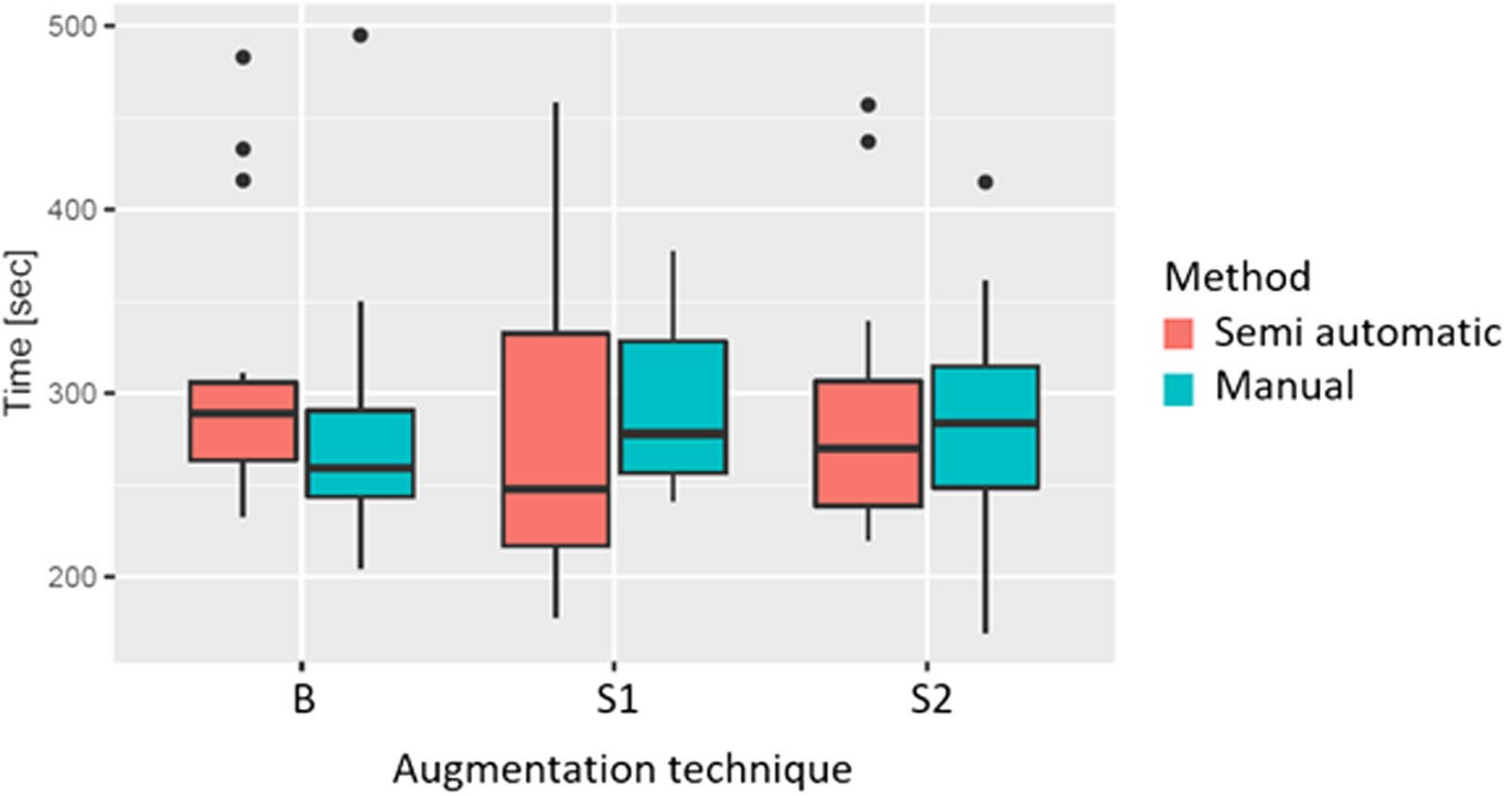
Time needed to measure the volume. Time needed to measure the volume for different augmentation techniques (B: block augmentation, S1: split-block augmentation, S2: shell) and comparison of the semi-automatic approach and the manual slice-by-slice annotation

The mean time to measure a bone block using the manual method was 276.1 ± 63.12 s, while using the semiautomatic method, it was 302.63 ± 67.88 s. Measuring the split block augmentation S1 took 294.25 ± 45.70 s using the manual method and 275.68 ± 77.57 s with the semiautomatic approach. Finally, the mean measuring time for augmentation technique S2 was 288 ± 53.41 s for the manual method and 285.68 ± 66.82 s for the semiautomatic approach. These differences in the mean time required between the two methods were not statistically significant (*p* > 0.05, Table 2; Fig. 8).

**Table 2:** Time needed for measurement for each augmentation technique

	Semi-automatic (*n* = 19) [s]	Manual (*n* = 20) [s]	Total (*n* = 39) [s]	*p* values (Wilcoxon two-sample signed-rank test)
Block	302.63 ± 67.88	276.1 ± 63.12	289.03 ± 65.99	0.432
Split block	275.68 ± 77.57	294.25 ± 45.70	285.21 ± 63.11	0.147
Shell	285.68 ± 66.82	288.00 ± 53.41	286.87 ± 59.52	0.520

Analysis times for the augmentation techniques, and the two measuring techniques (semi-automatic and manual). No statistically significant values with significance level alpha < 0.05

## Discussion

Various bone grafting procedures are used for treating alveolar bone defects. To reliably evaluate and compare different grafting procedures, the stability of the transplanted bone volume is a key indicator of a good grafting technique.

The current standard method used to evaluate the results of postoperative augmentation is generally based on either 2D or 3D measurements obtained by manual segmentation on individual slices of 3D data sets [11]. Concerning sinus floor augmentation, a comparison by volume based on segmentation of airspace volume has been shown to achieve accurate results and valuable insights concerning sinus floor elevation techniques [12]. This usually requires extensive manual annotation depending on the size of the augmented volume. Accordingly, the aim of this study was to establish an algorithm to measure the 3D volume of bone augmentation as effectively as the current gold standard based on currently used data formats.

The method of block augmentation from the retromolar region as a full block as well as a split block is an established intraoral bone grafting technique. These two techniques were chosen for this analysis, because the full block is a solid cortical block that has a dense structure on radiographs and can be well-segmented, whereas the split block is composed mainly of particulate bone and has only a very thin cortical shell to stabilize the particulate graft. This technique has a much less radiopaque appearance and is, therefore, not as easily differentiated as a full cortical block. These two groups were purposely selected to test the feasibility and reliability of the semiautomated technique compared with the manual technique. In addition, the shell augmentation technique combines the less radiopaque appearance and particulate matter properties with a curved surface contour.

We show that our proposed method produces measurements which are statistically not different from the current gold standard. Furthermore, the slice-by-slice annotation as well as our new proposed method possessed excellent intra-rater reliability.

In this study, the time consumption between the two methods did not differ significantly. We want to emphasize that the semi-automatic approach is a completely new method of volume measurement based on intraoral-scan STL files. Our proposed algorithm reduces manual labour, especially tedious slice-by-slice annotation, but some user interaction, e.g., the definition of the ROI is still required. The current workflow includes currently time-consuming steps which could be automated in the future to increase efficiency. Notable steps which offer the most potential are the closing of the surface mesh to a volume mesh (step 3) as well as the definition of the ROI (step 4). We estimate that with dedicated software at least 50% of the time can be saved.

In this study, we evaluated our proposed algorithm against the current gold standard on a data set of 20 pig jaws; nonetheless, due to the sample size, bias cannot be excluded.

The effect on interobserver reliability, as well as possible time-saving improvements, should be evaluated in a further study.

Although intraoral scanners are not practical to use in the operating theatre and directly scanning the bone surface is not feasible for recall controls, intraoral scanners can export surface data directly to the STL file format and achieve good scanning precision [13]. In addition, blood in the surgical site can negatively affect the precision of scans. However, cone-beam CT or CT data can be easily converted into STL files by a segmentation process. The aim of this study was to evaluate the practicability of our novel measurement algorithm. Using optical scanned surfaces, it was possible to circumvent imprecisions of a segmentation algorithm (thresholds of data sets) as well as CT/CBCT scans [14]. A further study to evaluate this influence and extend the method to Digital Imaging and Communications in Medicine (DICOM) files is currently ongoing.

## Conclusion

In this in vitro setting, measurements based on optical scanning techniques using mesh data were used. Our novel measuring approach achieves comparable results to the current gold standard in 3D volume measurement.

## Data Availability

The data sets used and analysed in the presented study are available from the corresponding author on reasonable request.

## Abbreviations

TOST: Test of one-sided significance
NHST: Null hypothesis significance test
CBCT: Cone beam computed tomography
STL: Surface triangle language
ROI: Region of interest
ICP: Iterative closest point
ICC: Intraclass correlation coefficients
B: Block augmentation
S1: Split block augmentation
S2: Shell augmentation
DICOM: Digital imaging and communications in medicine

## References

[1] Jensen SS, Terheyden H (2009). Bone augmentation procedures in localized defects in the alveolar ridge: clinical results with different bone grafts and bone-substitute materials. Int J Oral Maxillofac Implants.

[2] Rocchietta I, Fontana F, Simion M (2008). Clinical outcomes of vertical bone augmentation to enable dental implant placement: a systematic review. J Clin Periodontol.

[3] Acocella A, Bertolai R, Colafranceschi M, Sacco R (2010). Clinical, histological and histomorphometric evaluation of the healing of mandibular ramus bone block grafts for alveolar ridge augmentation before implant placement. J Craniomaxillofac Surg.

[4] Li Y, Qiao S-C, Gu Y-X, Zhang X-M, Shi J-Y, Lai H-C (2019). A novel semiautomatic segmentation protocol to evaluate guided bone regeneration outcomes: a pilot randomized, controlled clinical trial. Clin Oral Impl Res.

[5] Smolka W, Eggensperger N, Carollo V, Ozdoba C, Iizuka T (2006). Changes in the volume and density of calvarial split bone grafts after alveolar ridge augmentation. Clin Oral Impl Res.

[6] Verdugo F, Simonian K, Smith McDonald R, Nowzari H (2010). Quantitation of mandibular symphysis volume as a source of bone grafting. Clin Implant Dent Relat Res.

[7] Arasawa M, Oda Y, Kobayashi T, Uoshima K, Nishiyama H, Hoshina H (2012). Evaluation of bone volume changes after sinus floor augmentation with autogenous bone grafts. Int J Oral Maxillofac Surg.

[8] Object Alignment Tool. github: github.com. https://github.com/patmo141/object_alignment.

[9] Kabsch W (1976). A solution for the best rotation to relate two sets of vectors. Acta Cryst A.

[10] Koo TK, Li MY (2016). A guideline of selecting and reporting intraclass correlation coefficients for reliability research. J Chiropr Med.

[11] Sbordone L, Toti P, Menchini-Fabris GB, Sbordone C, Piombino P, Guidetti F (2009). Volume changes of autogenous bone grafts after alveolar ridge augmentation of atrophic maxillae and mandibles. Int J Oral Maxillofac Surg.

[12] Gultekin BA, Borahan O, Sirali A, Karabuda ZC, Mijiritsky E (2016). Three-dimensional assessment of volumetric changes in sinuses augmented with two different bone substitutes. Biomed Res Int.

[13] Aragón MLC, Pontes LF, Bichara LM, Flores-Mir C, Normando D (2016). Validity and reliability of intraoral scanners compared to conventional gypsum models measurements: a systematic review. Eur J Orthod.

[14] Loubele M, Guerrero ME, Jacobs R, Suetens P, van Steenberghe D (2007). A comparison of jaw dimensional and quality assessments of bone characteristics with cone-beam CT, spiral tomography, and multi-slice spiral CT. Int J Oral Maxillofac Implants.

